# Supplementary material for: Less absorbed solar energy and more internal heat for Jupiter
Source: Nat Commun. 2018 Sep 13;9:3709. doi: 10.1038/s41467-018-06107-2 (PMC6137063; doi:10.1038/s41467-018-06107-2)
Supplement: Supplementary file 1 — Supplementary Information [file 41467_2018_6107_MOESM1_ESM.pdf]

Supplementary Information for

Less Absorbed Solar Energy And More Internal Heat For Jupiter

Liming Li[1*], X. Jiang[2], R. A. West[3], P. J. Gierasch[4], S. Perez-Hoyos[5], A. Sanchez-Lavega[5], L. N. Fletcher[6], J. J. Fortney[7], B. Knowles[8], C. C. Porco[8], K. H. Baines[3], P. M. Fry[9], A. Mallama[10], R. K. Achterberg[11], A. A. Simon[12], C. A. Nixon[12], G. S. Orton[3], U. A. Dyudina[13], S. P. Ewald[14], R. W. Schmude Jr.[15]

Affiliation:

[1] *Department of Physics, University of Houston, Houston, TX, USA.*

[2] *Department of Earth and Atmospheric Sciences, University of Houston, Houston, TX, USA.*

[3] *Jet Propulsion Laboratory, California Institute of Technology, Pasadena, CA, USA.*

[4] *Department of Astronomy, Cornell University, Ithaca, NY, USA.*

[5] *Departamento de Fisica Aplicada I, Escuela de Ingenieria UPV/EHU, Bilbao, Spain*

[6] *Department of Physics and Astronomy, University of Leicester, Leicester, UK.*

[7] *Department of Astronomy and Astrophysics, University of California, Santa Cruz, CA, USA.*

[8] *CICLOPS/Space Science Institute, Boulder, Colorado, USA.*

[9] *Space Science and Engineering Center, University of Wisconsin-Madison, Madison, WI, USA.*

[10] *Department of Mathematics and Statistics, University of Maryland, College Park, MD, USA.*

[11] *Department of Astronomy, University of Maryland, College Park, MD.*

[12] *NASA Goddard Space Flight Center, Greenbelt, MD, USA.*

[13] *Space Science Institute, Boulder, CO, USA.*

[14] *Division of Geological and Planetary Sciences, Caltech, Pasadena, CA, USA.*

[15] *Gordon State College, Barnesville, GA.*

- To whom all correspondence should be addressed. E-mail: lli7@central.uh.edu

This file includes:

Supplementary Tables 1-2

Supplementary Figures 1-19

Supplementary References

Supplementary Table 1. Observational Data Sets For Measuring Jupiter's Bond albedo.

Variable	Cassini Observations	Other Observations
Solar Spectral Irradiance (SSI)		SEE (0-120nm, 2002), UARS (120-420 nm, 2000-01), SORCE (420-2410 nm, 2003), and ASTM (2410-4000 nm, climatology)
Phase Integral	ISS (266-938 nm, 2000-01) and VIMS (350-4000 nm, 2000-01)	
Reflectance Spectra	ISS (266-938 nm, 2000-01) and VIMS (350-4000 nm, 2000-01)	IUE (125-195 nm, 1978-80), Aerobee rocket (210-300 nm, 1963-1964), ESO (300-1000 nm, 1993, 1995)

Note: The numbers in the parentheses are observational wavelengths and times. The full names of the abbreviations in the table are as follows: ISS is the Imaging Science Subsystem on the Cassini spacecraft[1], VIMS is the Visual and Infrared Mapping Spectrometers on the Cassini spacecraft[2], SEE is the Solar EUV Experiment (http://lasp.colorado.edu/lisird/see/level3/3_ssi.html), UARS is the Upper Atmosphere Research Satellite (http://lasp.colorado.edu/lisird/uars/uars_ssi/index.html), SORCE is the Solar Radiation and Climate Experiment (http://lasp.colorado.edu/lisird/sorce/sorce_ssi/index.html), ASTM is American Society for Testing and Materials (http://rredc.nrel.gov/solar/spectra/am1.5/), IUE is the International Ultraviolet Explorer[3], and ESO is European Southern Observatory[4, 5]. In addition, the data from Aerobee Rocket are based on the papers by Stecher[6, 7].

Supplementary Table 2. Effective Wavelengths Of The Cassini ISS Filters.

**Filter**	**Effective wavelength**		**Filter**	**Effective wavelength**	
	NAC	WAC		NAC	WAC
BL1	455 nm	463 nm	MT1	619 nm	-
GRN	569 nm	568 nm	MT2	727 nm	728 nm
RED	649 nm	647 nm	MT3	889 nm	890 nm
UV1	264 nm	-	CB1	CB1a 635 nm; CB1b 603 nm	-
UV2	306 nm	-	CB2	750 nm	752 nm
UV3	343 nm	-	CB3	938 nm	939 nm

Note: Effective wavelengths of the Cassini ISS filters are computed using the full system transmission function convolved with a solar spectrum[1]. There are more filters in the ISS NAC than the ISS WAC.

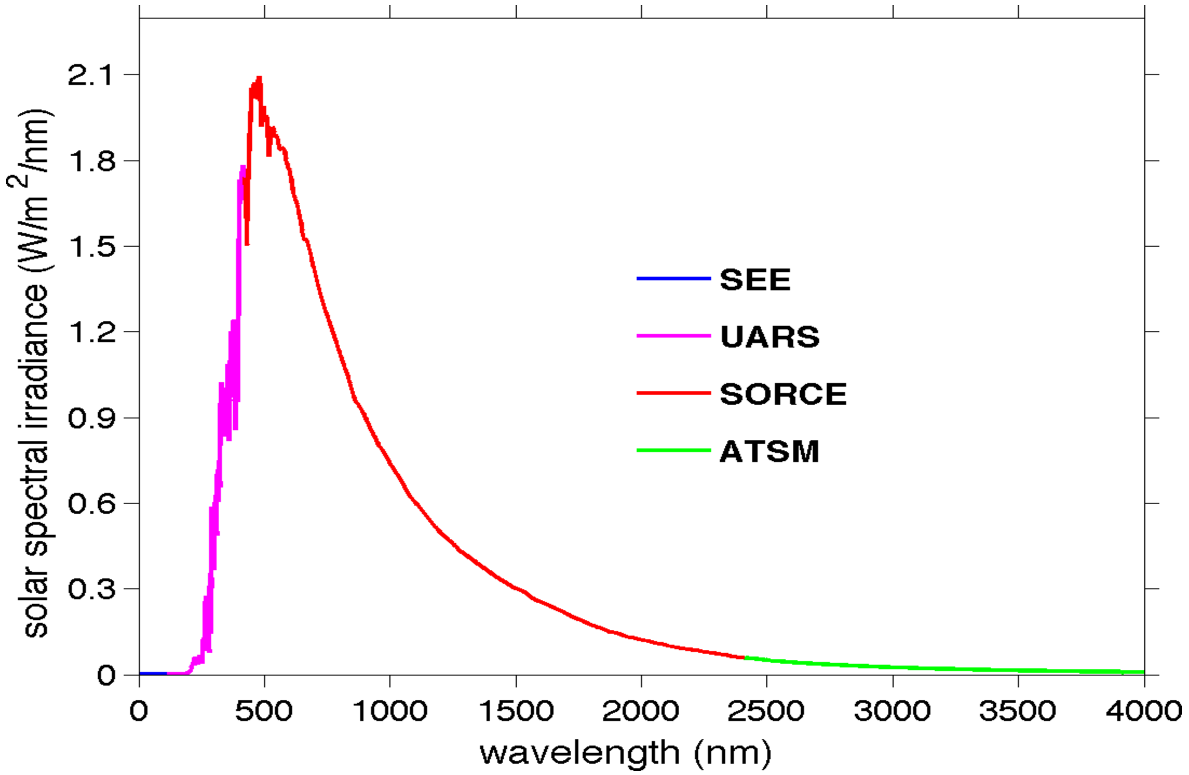

Supplementary Figure 1. The SSI in 2000-01 or the closet times from different data sets (see Supplementary Table 1).

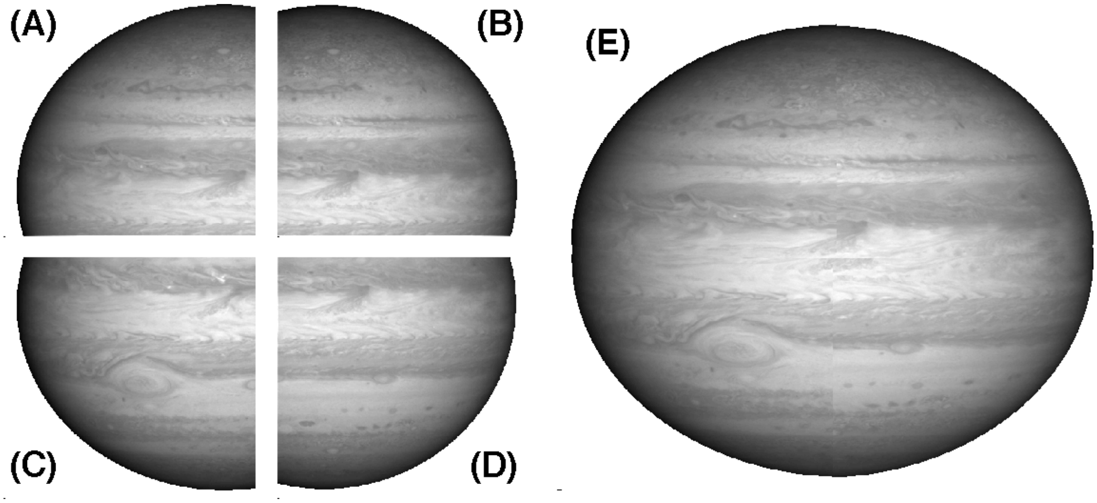

Supplementary Figure 2. Making a global image based on the four ISS images taken at the CB1 filter. The four quarterly images (panels a, b, c, and d) were taken on December 10, 2000 with a phase angle ~ 3.6° and a spatial resolution ~ 250 km/pixel. The four images were taken in basically the same viewing geometry with a quasi-simultaneous mode (time separation among the four images is ~ a few minutes), so they can be processed into a global image (panel e).

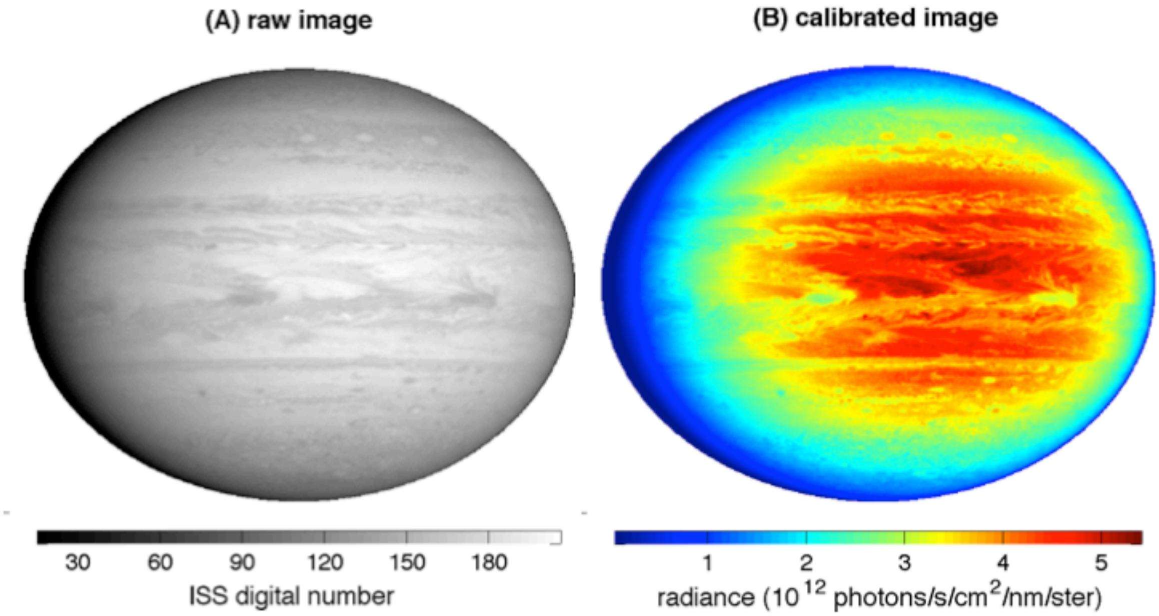

Supplementary Figure 3. Examples of raw ISS images and the calibrated images. The raw image was taken by the ISS CB2 filter on November 14, 2000 with a phase angle ~ 17.6° and a spatial resolution ~ 262 km/pixel. The calibration is conducted by the Cassini/ISS calibration software CISSCAL (version 3.8).

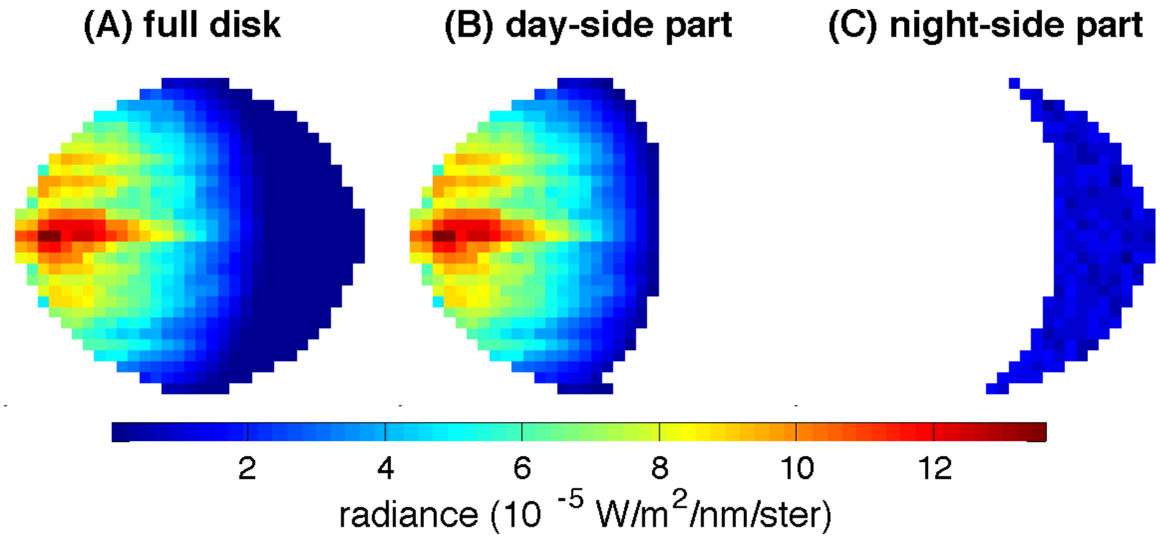

Supplementary Figure 4. Examples of VIMS calibrated images. The corresponding raw image was taken by the VIMS on December 30, 2000 with a phase angle ~ 63.3° and a spatial resolution ~ 4000 km/pixel. The VIMS took images from ~ 350 nm to ~ 5000 nm. The examples shown here have a wavelength ~2760 nm.

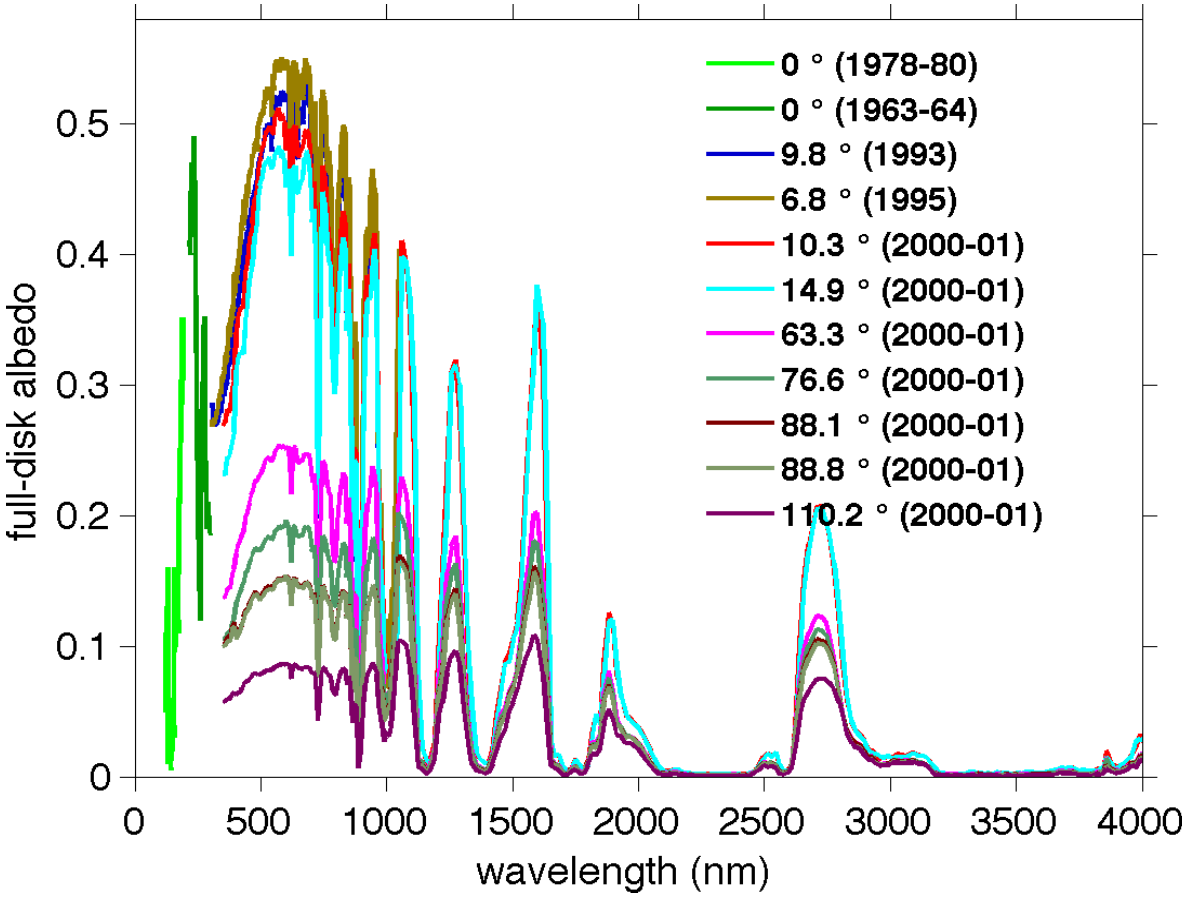

Supplementary Figure 5. Jupiter's albedo spectra at different phase angles from the Cassini VIMS observations in 2000-01 and other observations before 2000 (see Supplementary Table 1). The spectra from ~ 350 nm to ~ 4000 nm at seven phase angles (10.3°, 14.9°, 63.3°, 76.6°, 88. 1°, 88.2°, 110.2°) were recorded by the Cassini VIMS in 2000-01. The spectra from 125 nm to 195 nm were observed by the International Ultraviolet Explorer[3] in 1978-80 with a phase angle 0°. The spectra from 210 nm to 300 nm were recorded by Aerobee Rocket in 1963-64 with a phase angle 0° too[6, 7]. The spectra from 310 nm to 1050 nm were obtained by the European Southern Observatory in 1993 and 1995 with phase angles 9.8° and 6.8°, respectively[4, 5].

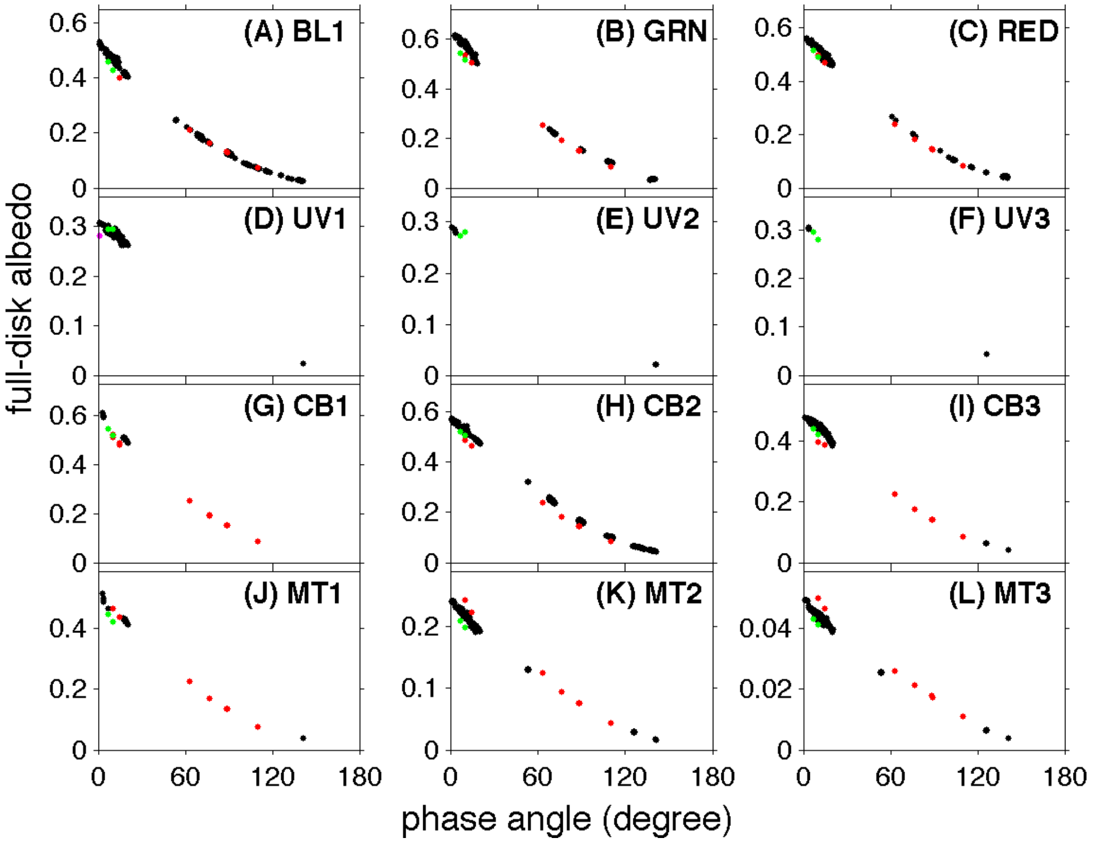

Supplementary Figure 6. Comparison of Jupiter's albedo between the Cassini ISS/VIMS observations and other observations at the 12 filters (i.e., wavelengths) covered by Cassini ISS (see Supplementary Table 2). Black and red dots represent the measurements from the Cassini ISS and VIMS, respectively. Green dots are from the European Southern Observatory[4, 5] and the magenta dot in panel D comes from the Aerobee Rocket[6, 7] (see Supplementary Table 1).

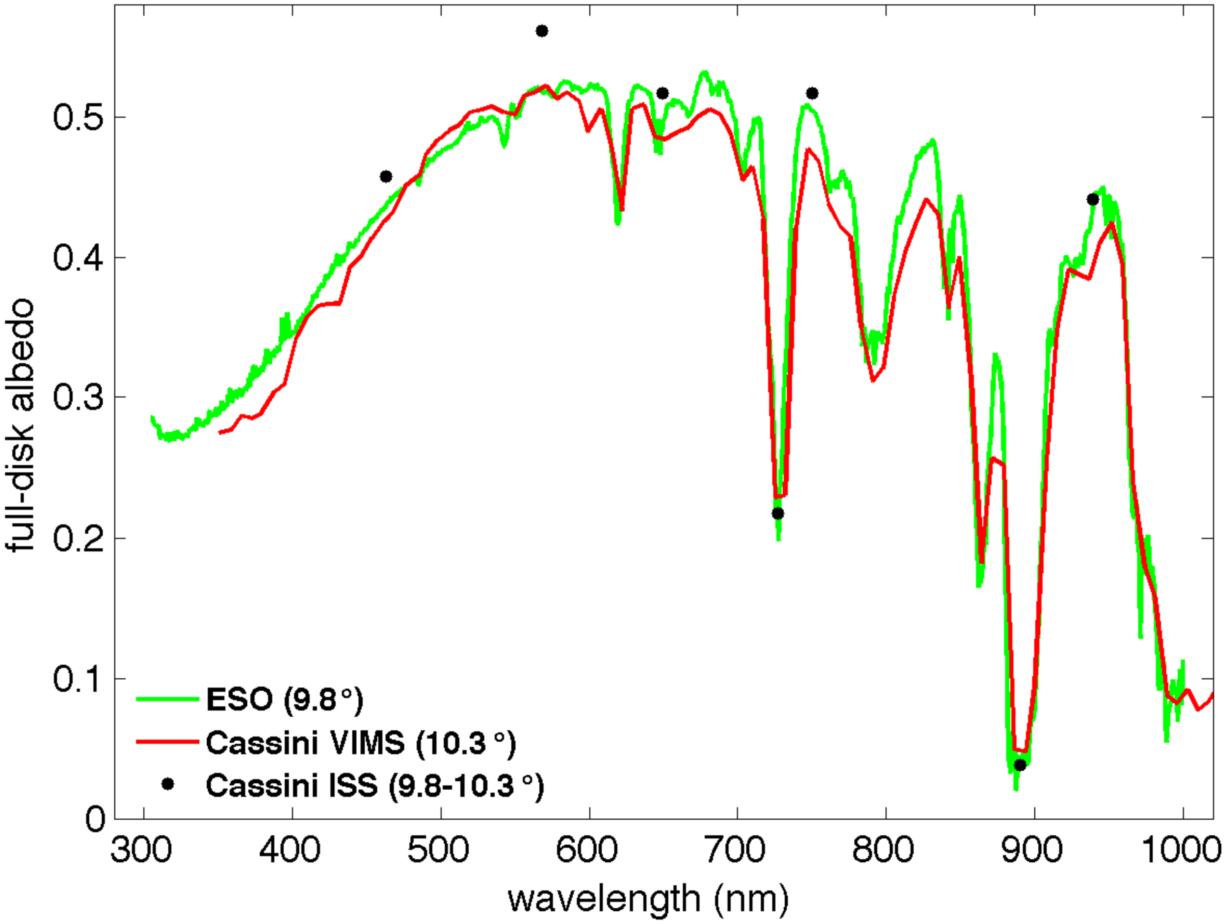

Supplementary Figure 7. Comparison of Jupiter's full-disk albedo among the European Southern Observatory (ESO), the Cassini/ISS, and the Cassini/CIRS at phase angles from 9.8° and 10.3°.

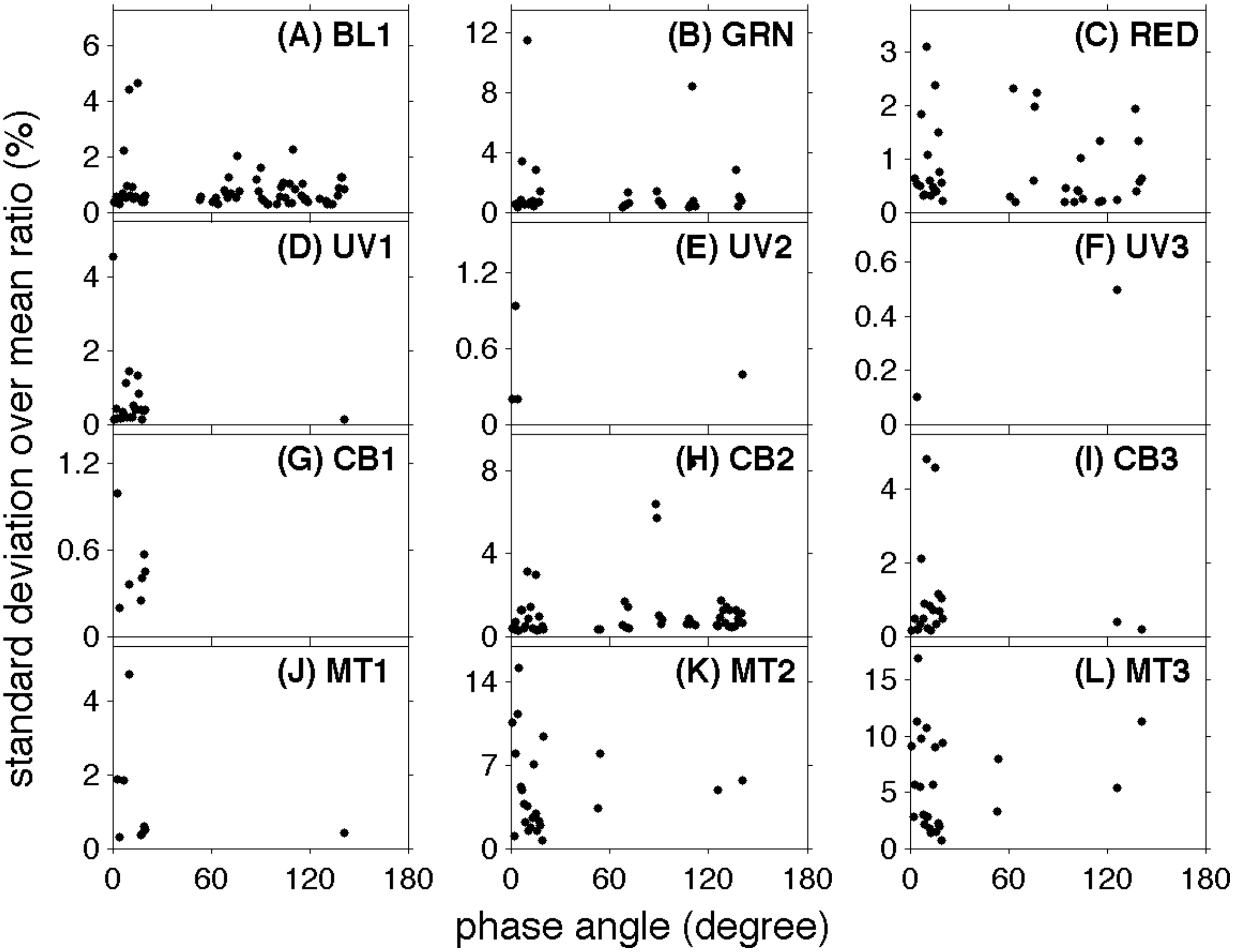

Supplementary Figure 8. Standard deviation of measurements for the phase angles with multiple measurements from the Cassini observations and other observations (refer to Supplementary Fig. 6)

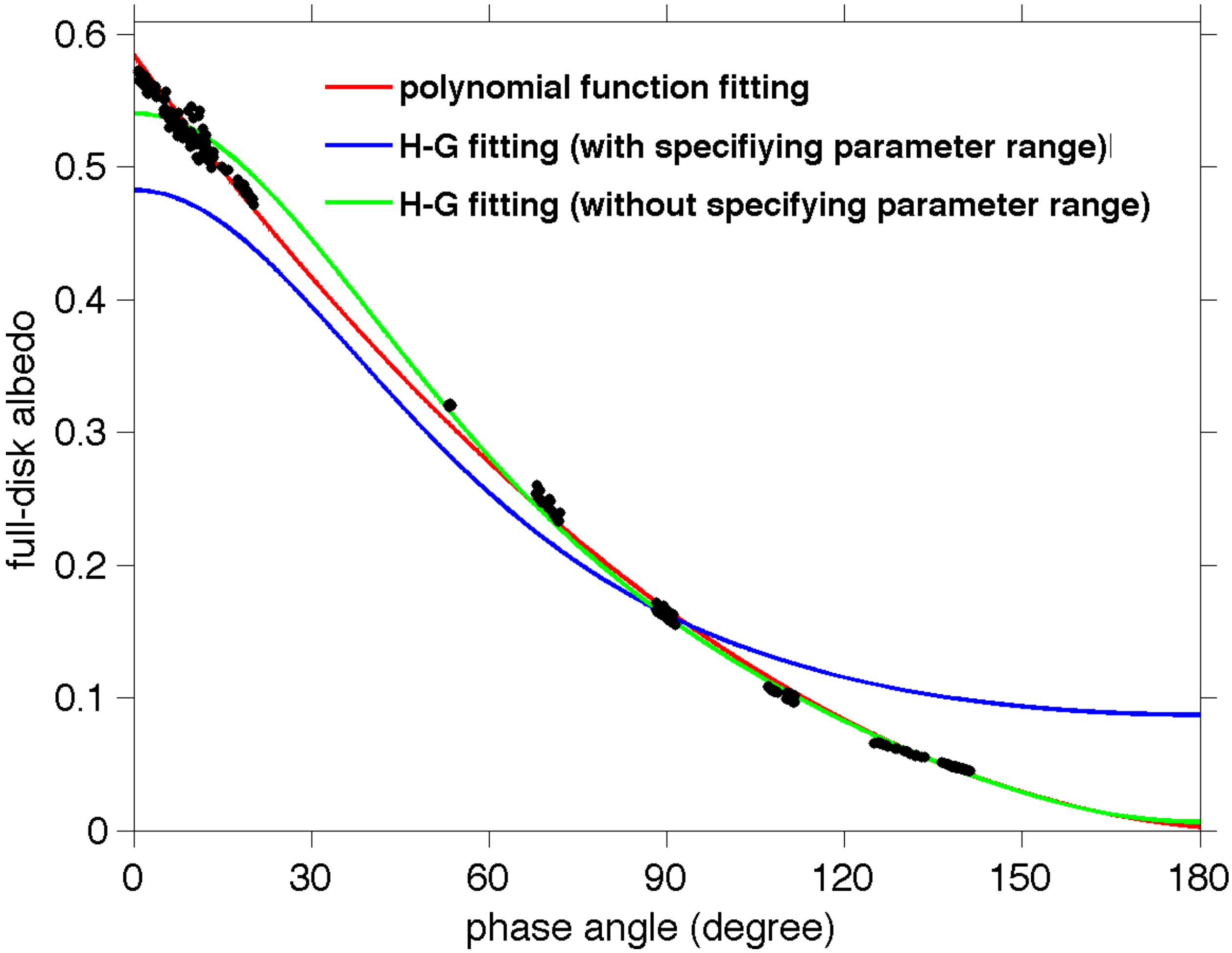

Supplementary Figure 9. Comparison of fitting among different functions for Jupiter's albedo recorded by the Cassini CB2 filter.

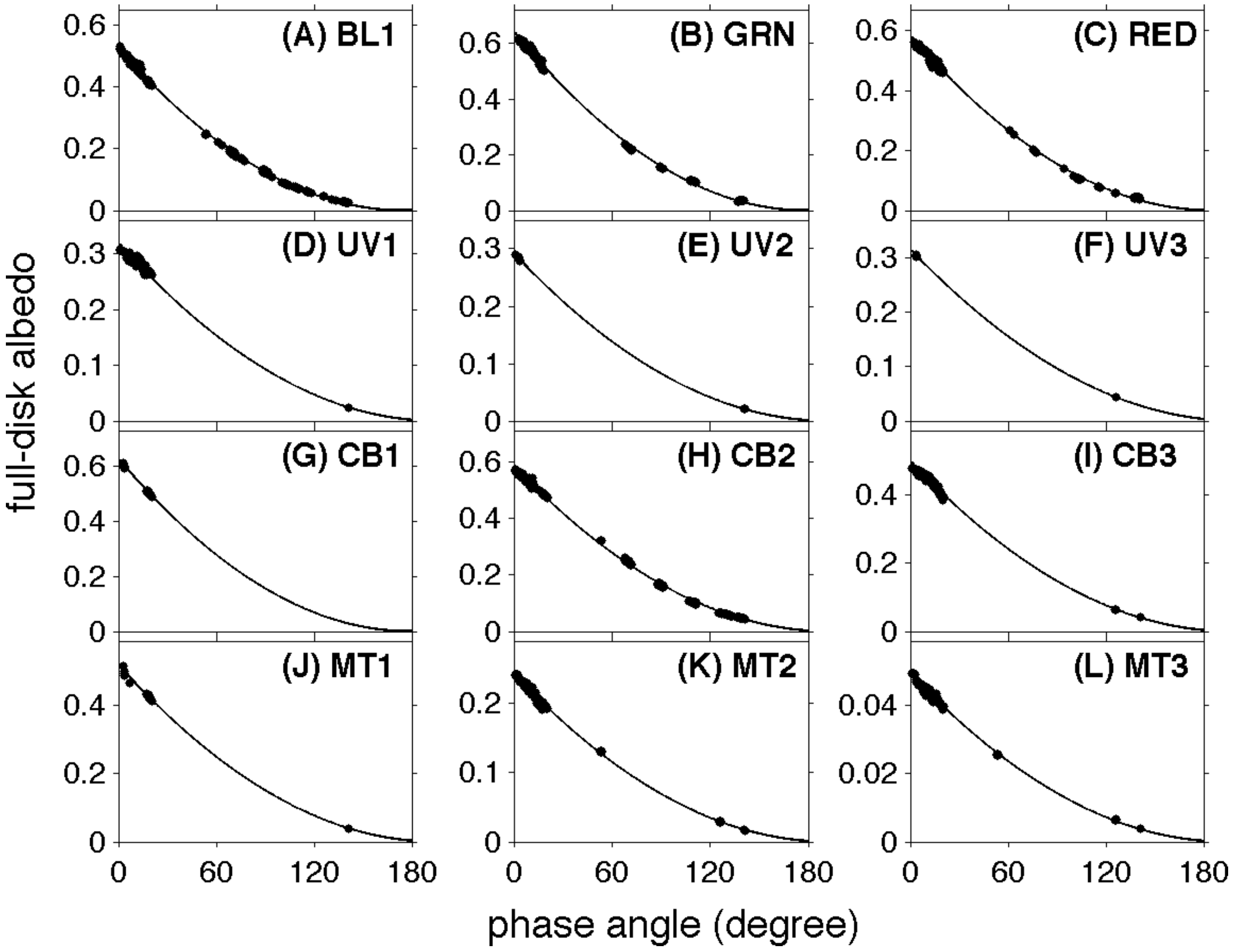

Supplementary Figure 10. Fitting the phase function of Jupiter's albedo recorded by the Cassini ISS 12 filters. Equation 6 in Methods is used to fit the observations with the least-squares method.

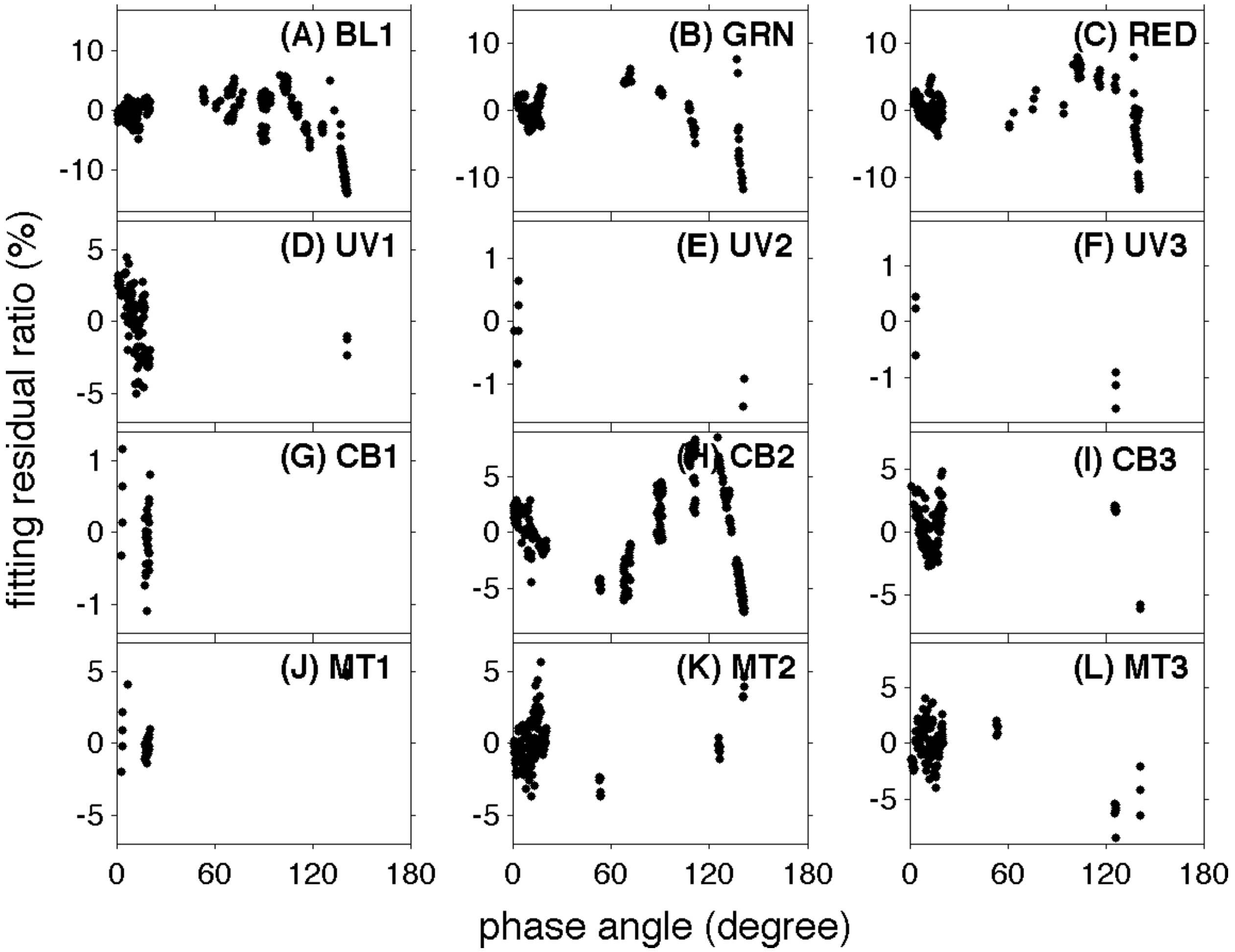

Supplementary Figure 11. Ratio between fitting residual and observational albedo. The fitting residual is defined as the difference between fitting albedo and the observational albedo, which is based on the results shown in Supplementary Fig. 10.

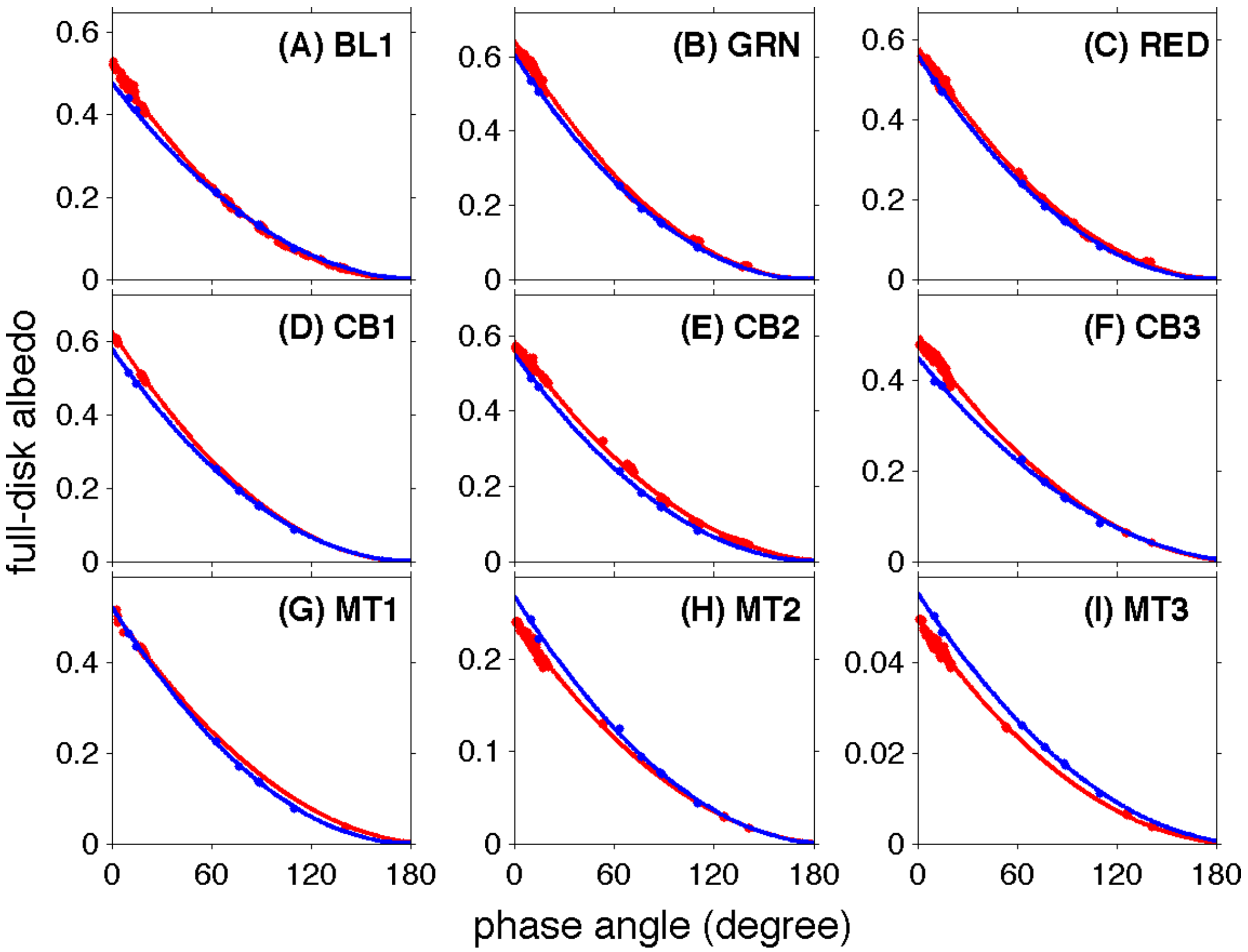

Supplementary Figure 12. Comparison of fitting between the ISS and VIMS observations. Red dots and lines are the ISS data and fitting results, respectively. Blue dots and lines are the VIMS data and fitting results, respectively. Note: the VIMS observations (~ 350-4000 nm) do not cover the three ultraviolet wavelengths in the Cassini ISS (see Supplementary Table 2).

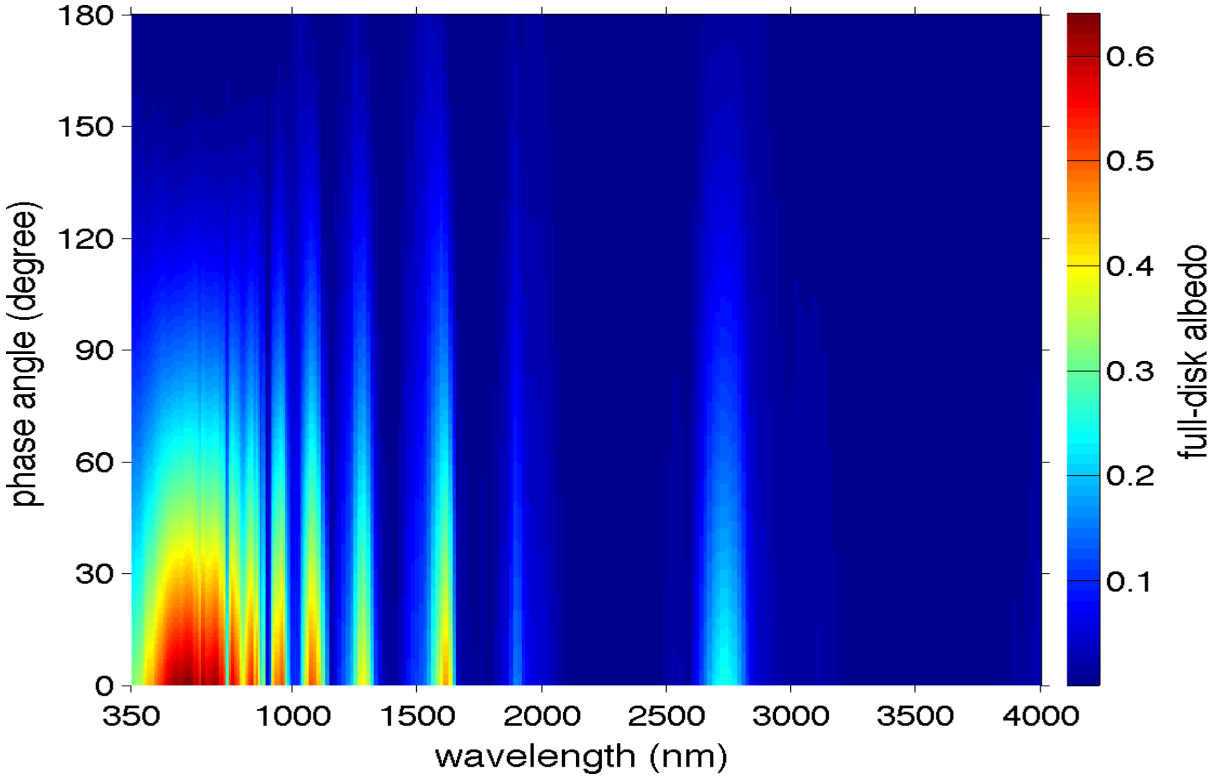

Supplementary Figure 13. Fitting the phase function of Jupiter's albedo recorded by the Cassini VIMS. Equation 6 in Methods is used to fit the VIMS observations from ~ 350 nm to ~ 4000 nm.

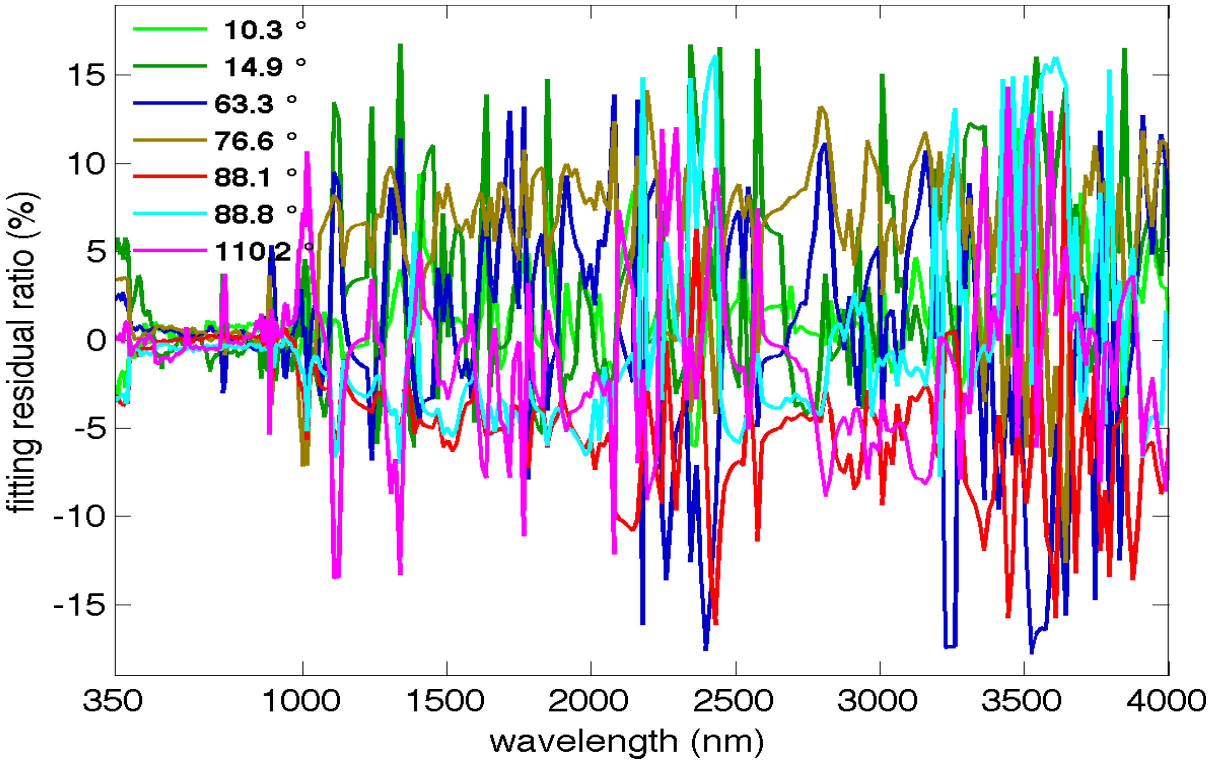

Supplementary Figure 14. Ratio between fitting residual and observed albedo. The fitting residual is defined as the difference between fitting albedo and the observed albedo, which is based on the results shown in Supplementary Fig. 13. High-quality VIMS observations were recorded in seven different phase angles (10.3°, 14.9°, 63.3°, 76.6°, 88. 1°, 88.2°, 110.2°), so the residual ratio is computed for the seven phase angles over the wavelength from 350 nm to 4000 nm.

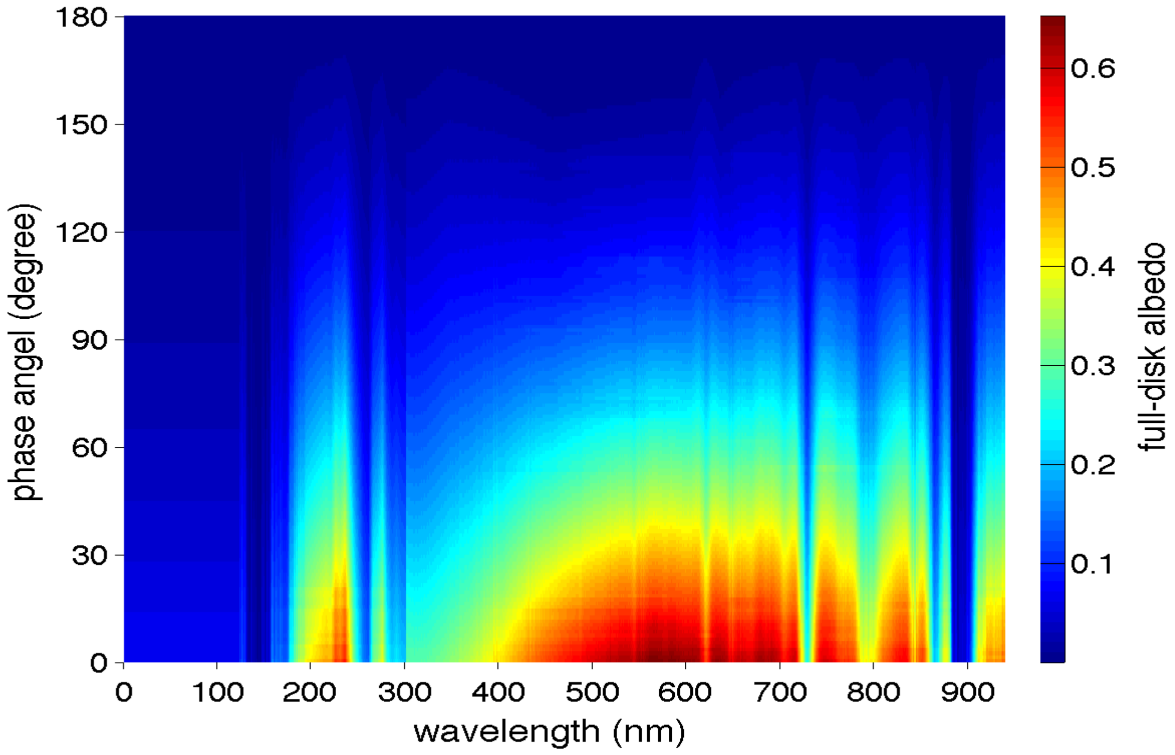

Supplementary Figure 15. Derived albedo in the 2-D domain of phase angle (0-180°) and wavelength (0-939 nm). The derived albedo is based on the ISS fitting phase functions in Fig. S10 and the reference albedo spectra at the available phase angles (see discussion in Methods section "Filling observational gaps in wavelength").

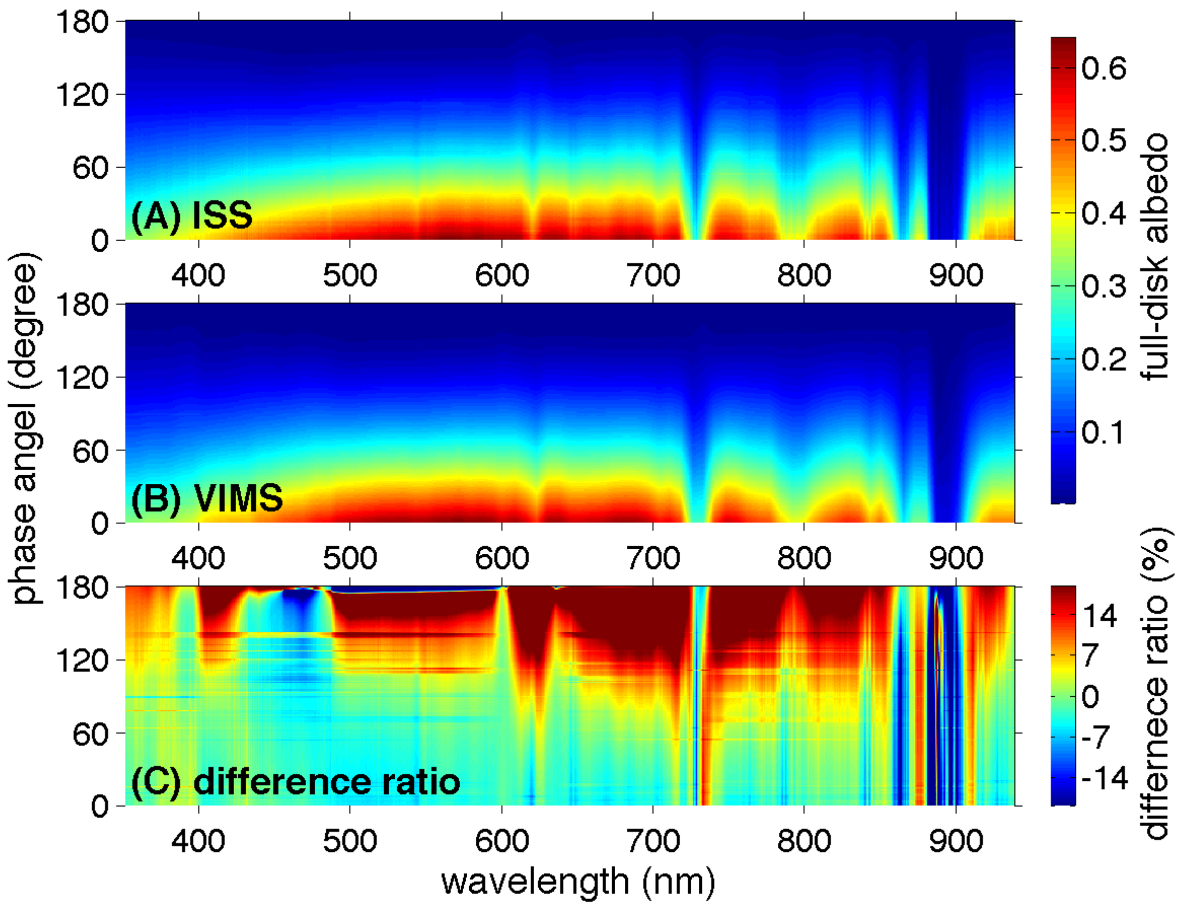

Supplementary Figure 16. Comparison of albedo between the ISS derived results and the VIMS fitting results for the overlap wavelengths (~ 350-939 nm) between the ISS and the VIMS. (a) The ISS derived albedo from Supplementary Fig. S15. (b) The VIMS fitting albedo from Supplementary Fig. 13. (c) Difference ratio. The difference ratio is defined as the ratio of the difference between the ISS and VIMS results over their mean value.

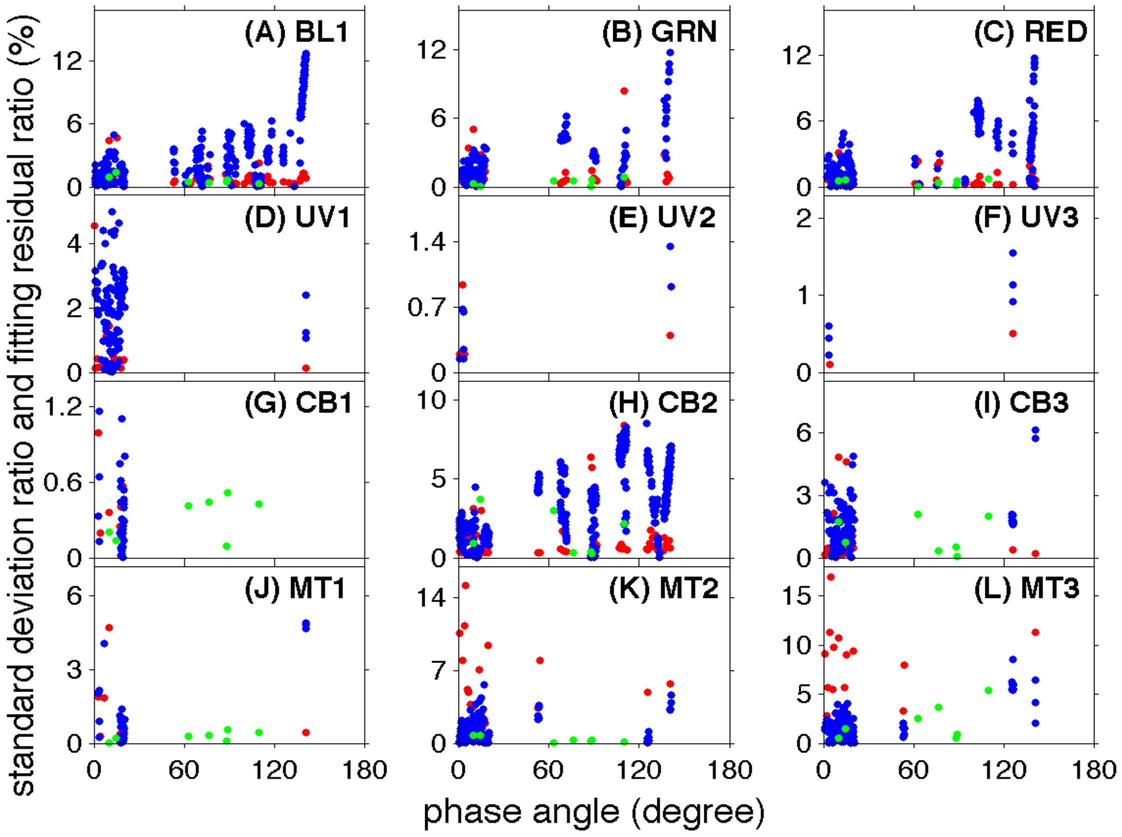

Supplementary Figure 17. Comparison between the standard deviation and the fitting residual. Red dots are the standard deviation ratios for the phase angles with multiple measurements from the Cassini and other observations, which are from Supplementary Fig. 8. Blue dots are the fitting residual ratios for the ISS fitting results, which are from Supplementary Fig. 11. Green dots are the fitting residual ratios for the VIMS observations at the seven phase angles (10.3°, 14.9°, 63.3°, 76.6°, 88.1°, 88.2°, 110.2°). The results of the VIMS fitting residual ratios (Supplementary Fig. 13 from 350 to 4000 nm) in the ISS 12 wavelengths are selected for the comparison. Please note that the blue and green dots are the absolute values of the fitting residual ratios for the ISS and VIMS observations (Supplementary Figs. 11 and 14) to be consistent with the positive standard deviation ratios (red dots).

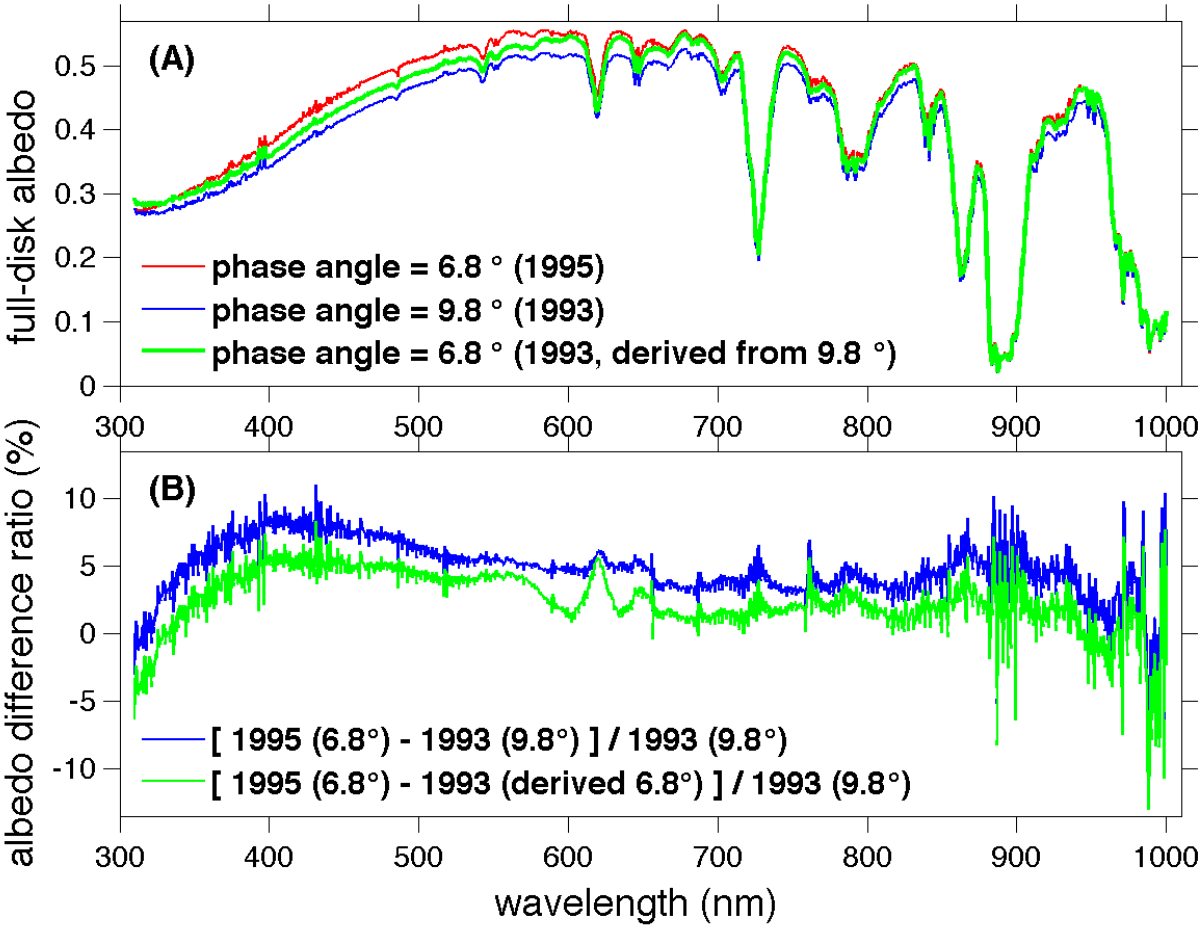

Supplementary Figure 18. Possible temporal variation of Jupiter's albedo. (a) Jupiter's albedo in 1993 (phase angle 9.8°)[4] and 1995 (phase angle 6.8°)[5]. In addition, we transform the results in 1993 with a phase angle 9.8° to a phase angle 6.8° based on the ISS phase functions (green line). (b) Difference ratio. Blue line is the difference ratio for the observations, in which the difference ratio is defined as the ratio of the albedo difference between 1993 and 1995 over the observed albedo in 1993. Green line is the difference ratio with the derived result in 1993 with a phase angle 6.8°, in which the difference ratio is defined as the ratio of the albedo difference between the observed result in 1995 (6.8°) and the derived result in 1993 (6.8°) over the observed albedo in 1993.

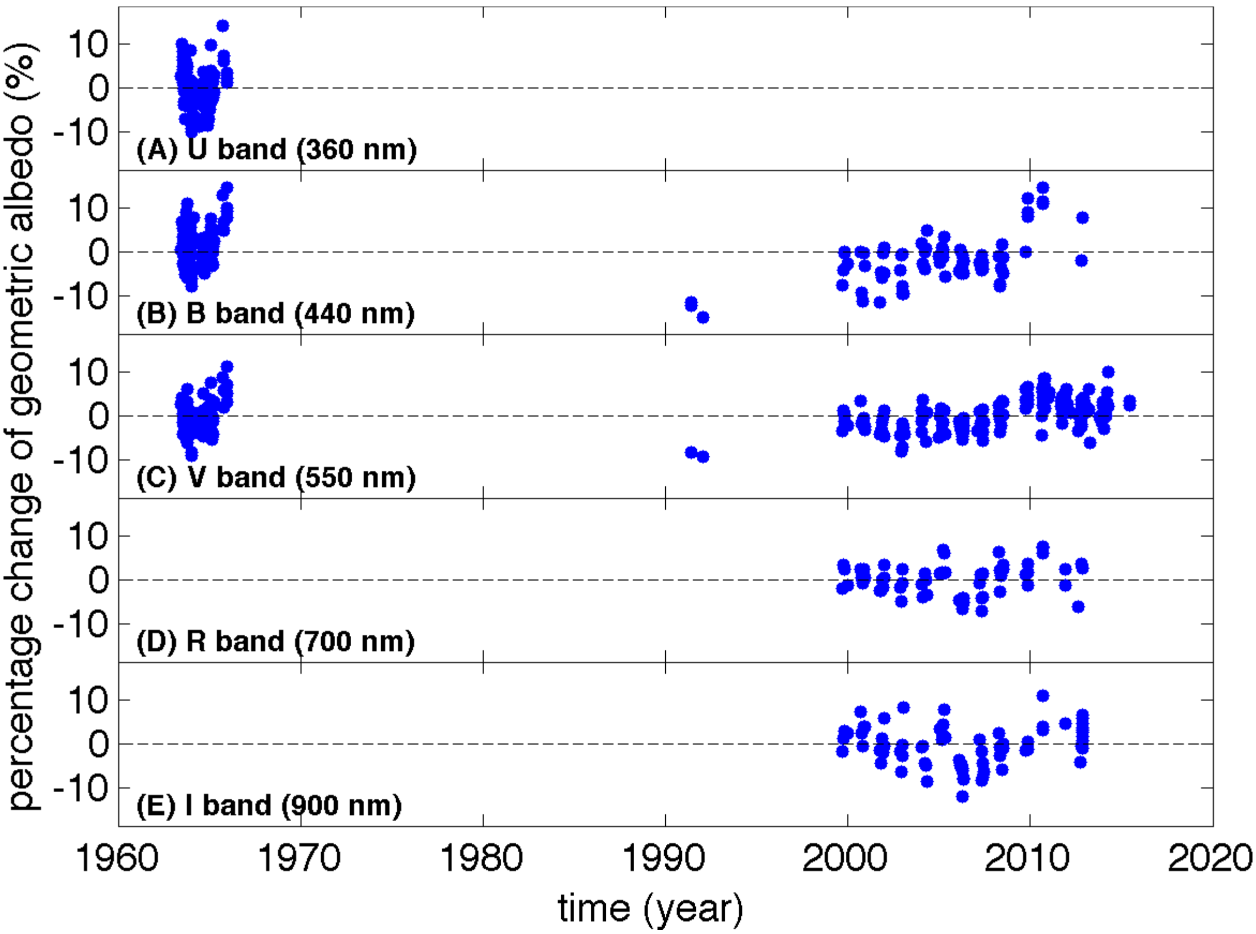

Supplementary Figure 19. Long-term variations of Jupiter's full-disk geometric albedo (1963-2015). Please note that the three data sets[8] analyzed here have some observational gaps during the period of 1963-2015, especially for the U filter.

Supplementary References

1. Porco, C. C. et al. Cassini Imaging Science: Instrument characteristics and anticipated scientific investigations at Saturn, *Space Sci. Rev.* **115**, 363-497 (2004).
2. Brown, R. H. et al. 2004. The Cassini visual and infrared mapping spectrometer (VIMS) investigation, *Space Sci. Rev.* **115**, 111-168 (2004).
3. Clarke, J. T., Moos, H. W. & Feldman, P. D. The far-ultraviolet spectra and geometric albedos of Jupiter and Saturn. *The Astrophysical Journal* **255**, 806-818 (1982).
4. Karkoschka, E. Spectrophotometry of the jovian planets and Titan at 300-to 1000-nm wavelength: The methane spectrum. *Icarus* **111**, 174-192 (1994).
5. Karkoschka, E. Methane, Ammonia, and Temperature Measurements of the Jovian Planets and Titan from CCD–Spectrophotometry. *Icarus* **133**, 134-146 (1998).
6. Stecher, T. P. January. An observation of Jupiter in the ultra-violet. In *Symposium-International Astronomical Union* **23**, 189-191 (Cambridge University Press, 1965).
7. Stecher, T. P. 1965. The Reflectivity of Jupiter in the Ultraviolet. *The Astrophysical Journal*
8. Mallama, A. & Schmude Jr, R. W. Cloud band variations and the integrated luminosity of Jupiter. *Icarus* **220**, 211-215 (2012).
